# Less is More: Perception as a fun way to Rich Minimalism

**DOI:** 10.1177/20416695221089678

**Published:** 2022-04-04

**Authors:** Claus-Christian Carbon, Sandra Utz, Vera M. Hesslinger

**Affiliations:** Department of General Psychology and Methodology, 14310 University of Bamberg, Bamberg, Germany; Research Group EPAEG (Ergonomics, Psychological Aesthetics, Gestalt), Bamberg, Germany; Bamberg Graduate School of Affective and Cognitive Sciences (BaGrACS), Bamberg, Germany

**Keywords:** perception, fun, insight, economics, rich minimalism, illusion, Umwelt, reality

## Abstract

Perceptual science is important to understand how humans and other animals perceive and experience scenes, objects and events. So, it is the essential science to predict how we construct reality and our Umwelt. We learn from perceptual phenomena that we only need a minimal amount of information to create rich worlds of imagination and perception. As such, perception is the perfect analogue to what we would like to call “Rich Minimalism” – the way to save resources while having even more fun as our brains complete the missing parts in a creative way. Here, we briefly mention three little examples from basic research to demonstrate the power of perception for creating efficiency, effectiveness, and economy while having great fun with the resulting minimalism.

Fundamental scientific efforts are important to understand nature, culture, ultimately: the human being, in order to create theories for predicting effects, interactions, and human behaviour. Perceptual science is particularly important to understand how humans and other animals perceive and experience scenes, objects, and events, and so it is the essential science to predict how we construct reality and our Umwelt. We learn from perceptual phenomena that we only need a minimal amount of information to create rich worlds of perceptual and imaginary experience. In this sense, perception is the perfect analogue to what we would like to call “Rich Minimalism” – a way to save resources while having even more fun as our brains complete the missing parts in a creative way. Here, we describe three little examples from basic research to demonstrate the power of perception to create efficiency, effectiveness, and economy while adding fun to the resulting minimalism.

Our first example is a device advertised as an advent wreath. Against all traditions, it comes with only one single candle. Typically, there would be four candles, representing the four advent Sundays. The designers, however, saved on three of them. They simply made use of the power of perceptual illusions, and provided three reflective glass panels that can be added to the wreath as needed (see Figure 1a): Each reflective panel will create one illusionary candle joining the one that is physically present. So, you can go from one candle, to two, three, and finally four, by adding one more reflective panel from the second advent Sunday onwards (see Figure 1b). You save resources, and you will enjoy the item by reflecting on the “trick” behind your magic candles. See more material plus a full video for a neat demonstration here: https://osf.io/axjvz/.

**Figure 1.**
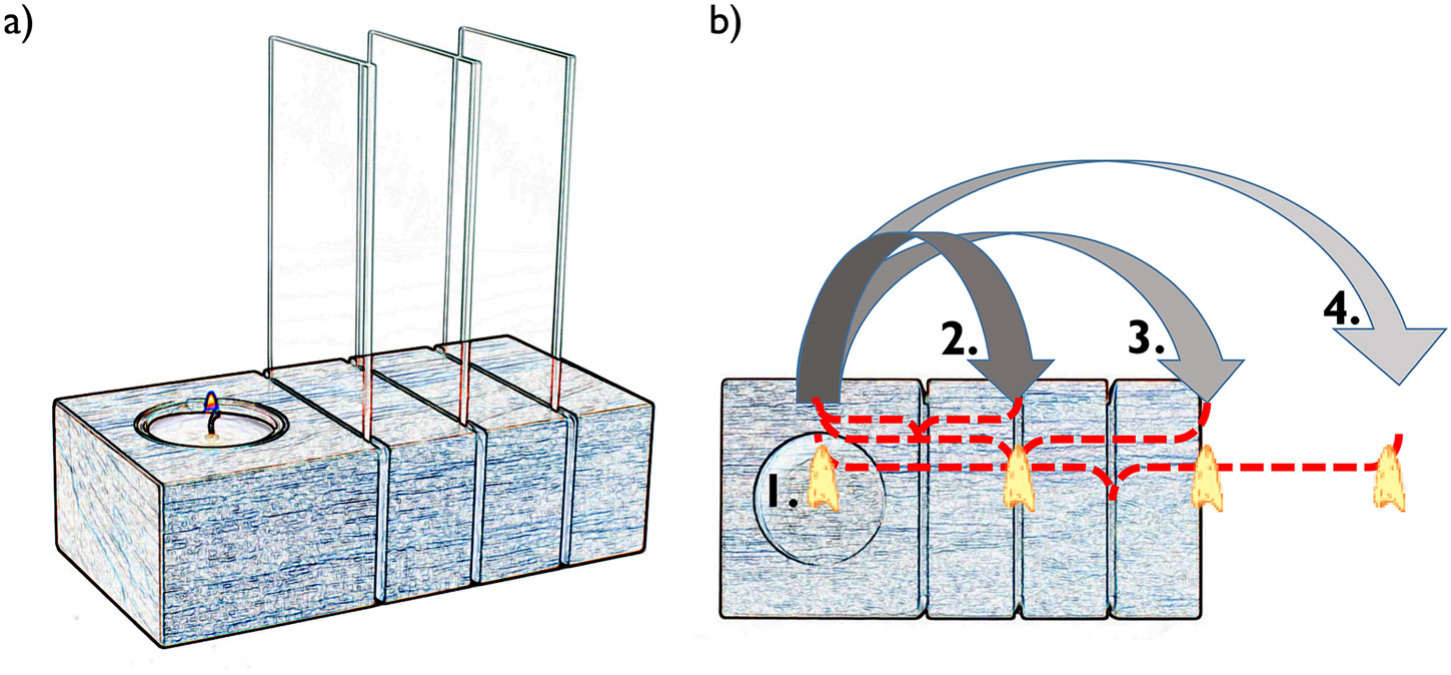
Schematic setup of the economic advent wreath which requires only one single candle but can easily give us the impression of 2, 3 or 4 candles being in action in order to assist the christmas countdown. a) structure of the device when ready for Xmas, b) functional principle of the device.

The second example is about colours. Usage of colour is often expensive, wastes resources, and pollutes the environment—plus: it is a lot of work to paint solid colours. If you want to minimize the usage of colour, yet still colourise your pictures, Gestalt psychology might help you out: Italian illusionist Pinna (1987), along with others (e.g., Pinna, Brelstaff, & Spillmann, 2001), came across a powerful colourisation effect based on figure-ground perception—the so-called Watercolour Illusion or Neon effect. If you add a fine coloured flank line to a coloured line encompassing a white area, this inner area is perceived in terms of a figure-ground segregation as a figure coloured in light hue similar to the flank line (see Figure 2). Making use of this effect, you will produce the impression of a fully painted area without having painted it—maximum effect with minimal resources. Whether Kandinsky was aware of the perceptual basis of the Watercolour Illusion is not relevant here; he obviously understood the principle quite well and applied it effectively. The closer inspection of his paintings reveals a great Aha-insight moment: The brain is the real creator; it “paints” the colours we see. The Watercolour Illusion, however, is just the tip of the iceberg: Colours do not exist physically, but only through our cognitive apparatus (von Helmholtz, 1910).

**Figure 2.**
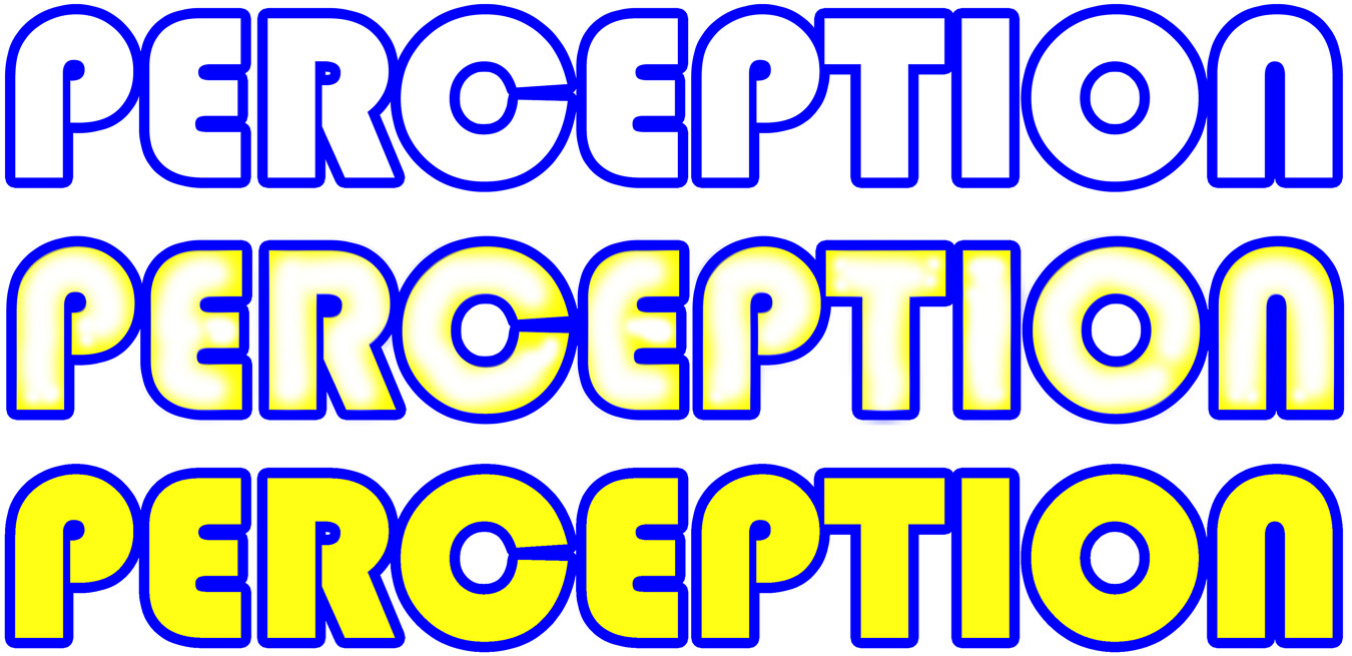
Demonstration of the Watercolour Illusion, created by CCC. Top row: A blue (RGB 0, 0, 255) outline of the word “Perception”; center row: the same blue outline along with a fine inner flank line of blurred yellow (RGB 255, 255, 0) creating the Watercolour Illusion of the inner parts of the characters which appear to be light yellow although perfectly white (RGB 255, 255, 255); bottom row: A comparison image to the center image—now a blue line together with a solid filling of the same yellow as the flank line in the center row shows saturated yellow characters with a blue outline.

With the third example, we would like to demonstrate how constructive our brains are in terms of creating Gestalt. Briefly glancing at Figure 3, you might observe a funny face because essential parts making up a face are present there (Morton & Johnson, 1991). Looking more closely, you have the chance to observe several different 2D as well as 3D figures. Our cognitive apparatus creates them by amodal completion (introduced by Michotte & Burke, 1951)—for further reading, see Gerbino (2020) and van Lier and Ekroll (2020). This critical mechanism allows us to identify objects even when complete visual information is lacking or when parts of an object are occluded (Nanay, 2018)—this is actually the case in most everyday situations. Amodal completion makes sense of a principally indeterminate visual input signal (Gregory, 1997). Missing pieces are automatically filled in anyway, are complemented and interpreted—we could even claim: the underlying processes open up possibilities for creative spaces. Determining the essential meaning is up to us, and the solution we take will give us a joyful moment back, called the Aesthetic Aha (Muth & Carbon, 2013) — actually, the recipient is actively involved in a deeper sense.

**Figure 3.**
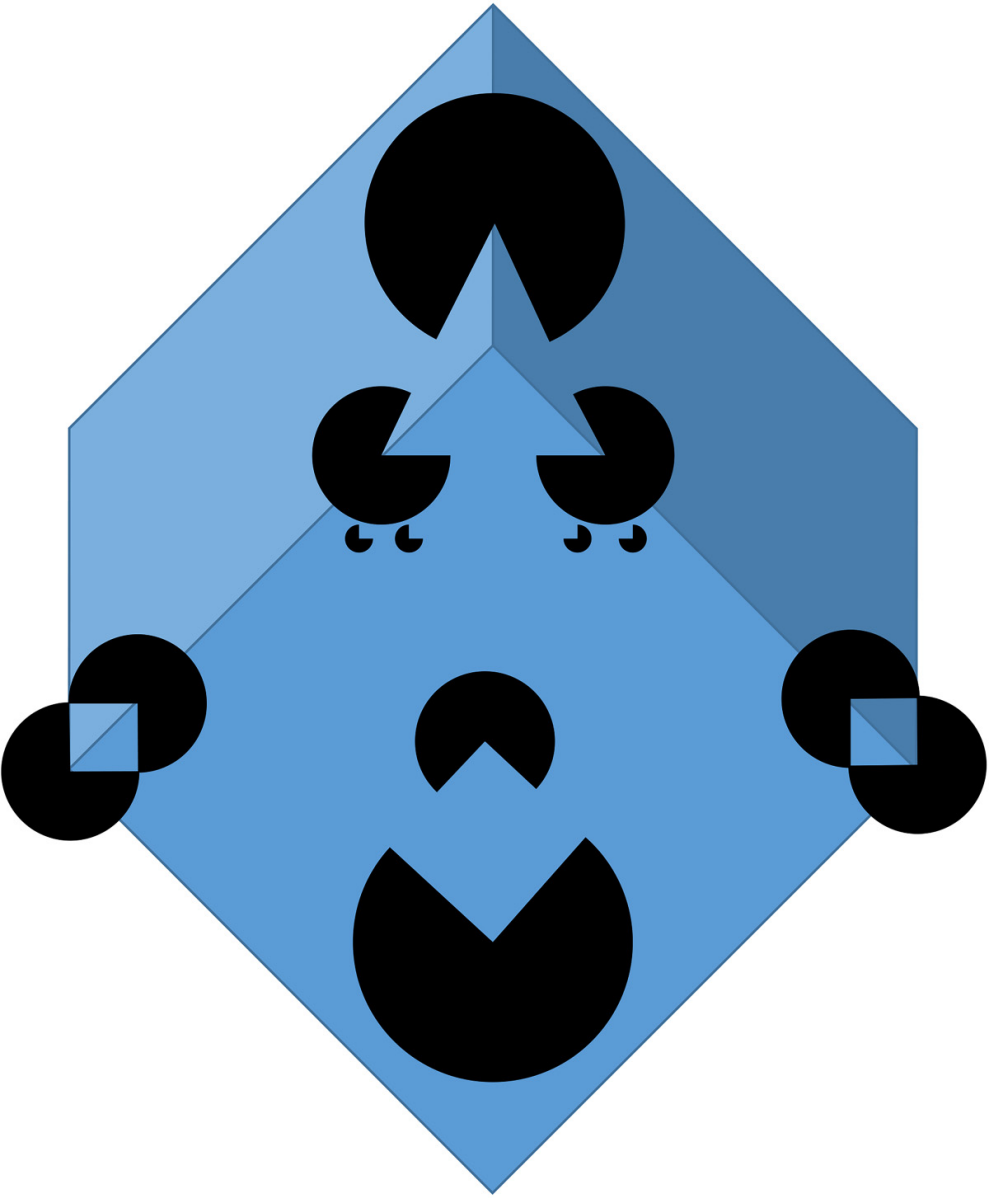
Illustration of the perceptual phenomenon of amodal completion and creating Gestalts which works for 2D as well as 3D—inspired by Kanizsa's famous triangle based on Ehrenstein's contour illusion, and created by CCC. The central object is a blueish cube which is partly occluded by black Pacman-like circular segments. The top 3 occluders create the illusion of a 3D object, actually a pyramid—when focusing on this pyramid, we will perceive clear edges even where no edges are physically present which separates the pyramid as figure from the ground. The lateral pairs of occluders create the illusion of squares which are divided by two different coloured halves—the 3D shadings of the underlying cube are now locally interpreted as different colour patches. The two center-bottom positioned occluders create the illusion of a slightly lighter blueish square with clear edges similar to Kanizsa's triangle. The whole configuration can be interpreted as a funny face with eyes made out of baby buggies.

The three examples we offer here are certainly only a very brief shortlist with selected highlights of perceptual science. We would like to ask our devoted readers to add their own experiences with perceptual phenomena, or to engage in exploring some new ones. The Oxford Compendium of Visual Illusions (Shapiro & Todorovic, 2018) is a great source of inspiration as is taking a look at popular books on optical illusions or participating in events like the “Illusion Night” or “ShowTime!” at the European Conference on Visual Perception (ECVP— https://ecvp.eu/) (Agostini, et al., 2021).

Gathering experiences with a variety of perceptual phenomena, especially so-called optical illusions, and informing ourselves about the mechanisms they are based on, we can playfully achieve an understanding of human perception and involved cognitive processing (Carbon, 2014). As our selection shows, this can also support efforts towards a more minimalistic life in which we use less material in a more intelligent way, but still create experiential abundance and have a lot more fun—or briefly: “Rich Minimalism”.

## References

[bibr1-20416695221089678] Agostini, T., Bertamini, M., Carbon, C. C., de la Malla, C., Domijan, D. Greenlee, M., … (2021). 43rd European conference on visual perception (ECVP) 2021 online. Perception, 50(1S), 1 − 244. 10.1177/03010066211059887 34989647

[bibr2-20416695221089678] Carbon C. C . (2014). Understanding human perception by human-made illusions. Frontiers in Human Neuroscience, 8(566), 1–6. 10.3389/fnhum.2014.00566 25132816 PMC4116780

[bibr3-20416695221089678] Gerbino W . (2020). Amodal completion revisited. I-Perception, 11(4), 1–26. 10.1177/2041669520937323 PMC7466902 32944209

[bibr4-20416695221089678] Gregory R. L. (1997). Eye and brain. Princeton University Press.

[bibr5-20416695221089678] Michotte A. Burke L. (1951). Une nouvelle énigme de la psychologie de la perception: Le “donné amodal” dans l’experience sensorielle [A new enigma in the psychology of perception: the “amodally given” in the sensory experience]. Paper presented at the 13th International Congress of Psychology, Stockholm, Sweden.

[bibr6-20416695221089678] Morton J. Johnson M. H . (1991). CONSPEC And CONLEARN: A two-process theory of infant face recognition. Psychological Review, 98(2), 164–181. 10.1037/0033-295x.98.2.164 2047512

[bibr7-20416695221089678] Muth C. Carbon C. C . (2013). The Aesthetic Aha: On the pleasure of having insights into Gestalt. Acta Psychologica, 144(1), 25–30. 10.1016/j.actpsy.2013.05.001 23743342

[bibr8-20416695221089678] Nanay B . (2018). The importance of amodal completion in everyday perception. i-Perception, 9(4), 2041669518788887–2041669518788887. 10.1177/2041669518788887 30109014 PMC6083800

[bibr9-20416695221089678] Pinna B. (1987). Un effetto di colorazione [A coloring effect]. Paper presented at Il laboratorio e la città. XXI Congresso degli Psicologi Italiani Milano.

[bibr10-20416695221089678] Pinna B. Brelstaff G. Spillmann L . (2001). Surface color from boundaries: A new ‘watercolor’ illusion. Vision Research, 41(20), 2669–2676. 10.1016/S0042-6989(01)00105-5 11520512

[bibr11-20416695221089678] Shapiro A. G. Todorovic D . (Eds.). (2018). The Oxford compendium of visual illusions. Oxford University Press.

[bibr12-20416695221089678] van Lier R. Ekroll V . (2020). A conceptual playground between perception and cognition: Introduction to the special issue on amodal completion. I-Perception, 11(4), 1–4. 10.1177/2041669520939108 PMC7343371 32685127

[bibr13-20416695221089678] von Helmholtz H. (1910). Handbuch der physiologischen Optik. (3rd ed.). Leopold Voss.

